# Pirfenidone affects human cardiac fibroblast proliferation and cell cycle activity in 2D cultures and engineered connective tissues

**DOI:** 10.1007/s00210-023-02421-9

**Published:** 2023-02-17

**Authors:** Friederike Elisabeth Ugi Meyer, Gabriela Leao Santos, Thao Phuong Doan, Alisa Nicole DeGrave, Bastian Bues, Susanne Lutz

**Affiliations:** 1 Institute of Pharmacology and Toxicology, University Medical Center, Goettingen, Germany; 2 Randall Centre for Cell and Molecular Biophysics, Kings College London, London, UK; 3 DZHK (German Centre for Cardiovascular Research) Partner Site, Goettingen, Germany

**Keywords:** Cardiac fibrosis, Pirfenidone, Human cardiac fibroblasts, Engineered connective tissues, Anti-fibrotic drugs

## Abstract

The anti-fibrotic drug pirfenidone (PFD) is currently in clinical testing for the treatment of heart failure with preserved ejection fraction; however, its effects on human cardiac cells have not been fully investigated. Therefore, we aimed to characterize the impact of PFD on human cardiac fibroblasts (CF) in 2D culture as well as in 3D-engineered connective tissues (ECT). We analyzed proliferation by automated cell counting and changes in signaling by immunoblotting. We generated ECT with different geometries to modify the cellular phenotype and investigated the effects of PFD on cell number and viability as well as on cell cycle activity. We further studied its effect on ECT compaction, contraction, stiffening, and strain resistance by ECT imaging, pole deflection analysis, and ultimate tensile testing. Our data demonstrate that PFD inhibits human CF proliferation in a concentration-dependent manner with an IC_50_ of 0.43 mg/ml and its anti-mitogenic effect was further corroborated by an inhibition of MEK1/2, ERK1/2, and riboprotein S6 (rpS6) phosphorylation. In ECT, a lower cell cycle activity was found in PFD-treated ECT and fewer cells resided in these ECT after 5 days of culture compared to the control. Moreover, ECT compaction as well as ECT contraction was impaired. Consequently, biomechanical analyses demonstrated that PFD reduced the stiffness of ECT. Taken together, our data demonstrate that the anti-fibrotic action of PFD on human CF is based on its anti-mitogenic effect in 2D cultures and ECT.

**Supplementary Information:**

The online version contains supplementary material available at 10.1007/s00210-023-02421-9.

## Introduction

Cardiac fibrosis is part of the detrimental remodeling process of the diseased heart. It is mainly driven by resident cardiac fibroblasts (CF), which become activated in response to chronic stress or acute injury and undergo transdifferentiation into the diseased myofibroblast phenotype. Once initiated, cardiac fibrosis self-propagates by a complex vicious circle involving the phenotypic shift of CF and the remodeling of the extracellular matrix (ECM) (Frangogiannis 2021). Nowadays, the most important strategies followed in anti-fibrotic therapy in heart disease involve a blockade of the renin–angiotensin–aldosterone system (Chai and Danser 2006; Fang et al. 2017); however, this is not sufficient to halt or reverse cardiac fibrosis. Therefore, new therapeutic options are needed and pirfenidone (PFD) is discussed as one promising anti-fibrotic drug (Aimo et al. 2022).

PFD is an orally available pyridinone derivative that has been approved for the treatment of idiopathic pulmonary fibrosis (IPF) in Europe since 2011. In 2017, a randomized, double-blind, placebo-controlled phase 2 study was initiated by Manchester University to test the efficacy and safety of PFD in patients with heart failure with preserved left ventricular ejection fraction (HFpEF) (trial name: PIROUETTE, NCT02932566) (Lewis et al. 2019). The first presented results demonstrated a modest reduction in the extracellular volume after 52 days for the PFD treatment group (Lewis et al. 2021).

The idea of using PFD for anti-fibrotic therapy in heart disease is obvious since all fibrotic diseases are thought to involve similar mechanisms. This includes, as mentioned above, the activation of tissue-resident fibroblasts by mechanical and biochemical cues. As especially TGF-β is in focus here, substances that interfere with this cytokine and its down-stream signaling are of high interest (Shi et al. 2022). In line, one discussed mechanism of action for PFD is an interference with the TGF-β signaling in (myo)fibroblasts (Aimo et al. 2022). However, the exact molecular target of PFD is not known, making it necessary to carefully investigate its effects in each disease context.

With respect to cardiac fibrosis, the anti-fibrotic action of PFD was demonstrated by diverse cardiac in vivo models, including pressure overload and myocardial infarction (Wang et al. 2013; Yamagami et al. 2015; Li et al. 2017, 2022). In 2D cultures of rodent CF, PFD inhibited TGF-β-induced transcriptional effects as well as proliferation, contraction, and migration (Shi et al. 2011; Yamagami et al. 2015). For 2D cultures of human CF, it was demonstrated that PFD moderately reduced the TGF-β1-induced ERK1/2 phosphorylation (Widjaja et al. 2021). However, it had no effect in a high content screen in which collagen 1 and α-smooth muscle actin (SMA) imaging served as a readout for the fibrotic status of CF (Palano et al. 2020). Likewise, inconclusive results on the anti-fibrotic properties of PFD were also found when engineered tissues containing CF were used. In rat CF-collagen discs, PFD reduced disc contraction (Shi et al. 2011). In two studies with heterocellular tissues containing human CF and inducible pluripotent stem cell-derived cardiomyocytes (iPSC-CM), PFD consistently reduced the expression of the fibrosis-associated gene periostin (POSTN) (Mastikhina et al. 2020; Bracco Gartner et al. 2022). However, in all 3D models, the observed effects of PFD were rather distinct and did not reflect a general inhibition of fibrotic processes, as SMA expression was either downregulated, unaffected, or even upregulated in response to PFD. Moreover, the postulated interference of PFD with the TGF-β pathway was inconsistently found (Mastikhina et al. 2020; Widjaja et al. 2021; Bracco Gartner et al. 2022).

As none of the 3D studies investigated the effect of PFD on CF cell cycle activity and viability, albeit PFD is a documented inhibitor of fibroblast proliferation (Shi et al. 2011; Conte et al. 2014; Cui et al. 2020), our aim was to investigate the anti-mitogenic effects of PFD on human CF in 2D cultures and 3D engineered connective tissues (ECT). Further, we tested PFD’s anti-fibrotic properties by determining the biomechanical properties of the ECT.

## Material and methods

### Material

Plasticware was obtained from Sarstedt, Nunc, and Greiner. PFD was purchased from MedChemExpress and TGF-β1 from Peprotech. Antibodies were obtained from Sigma-Aldrich (anti-α-smooth muscle actin A5228, anti-mouse IgG-HRP A9044, anti-rabbit IgG-HRP A9169), Invitrogen (anti-mouse IgG-Alexa Fluor 488 A1100), Cell Signaling Technologies (P-SMAD2 (Ser465/467), 3108, P-SMAD3 (Ser423/425), 9520, SMAD2 5339, SMAD3 9523, SMAD2/3 8685, P-p44/42 MAPK (ERK1/2) 9101, p44/42 MAPK (ERK1/2) 9102, P-MEK1/2 9121, MEK1/2 9122, P-rpS6 5301, Akt substrate 9614), and Santa Cruz (β-Tubulin, sc-58886). For fluorescence labeling of cells, wheat germ-agglutinin (WGA)-Alexa Fluor 488 conjugate (Thermo Fisher Scientific), TRITC-phalloidin (Sigma-Aldrich), Alexa Fluor 633-phalloidin (Invitrogen), DAPI (Sigma-Aldrich), propidium iodide (Sigma-Aldrich), and Hoechst33342 (Thermo Fisher Scientific) were used.

### Cells and cell culture

Normal human CF (ventricle, male donor, Lonza) and tsA201 cells (human embryonic kidney, immortalized by SV-40 large T antigen, Sigma-Aldrich) were used for the performed study.

Human CF cells were cultured in fibroblast growth medium-3 (FGM-3, cat. number C-23130, Promocell) composed of Basal Medium 3, 10% fetal calf serum, 1 ng/ml human basic fibroblast growth factor, and 5 µg/ml human insulin as well as additionally containing 100 U/ml penicillin and 100 µg/ml streptomycin (Thermo Fisher Scientific). The tsA201 cells were cultured in DMEM growth medium containing 4.5 g/l glucose, GlutaMAX (Thermo Fisher Scientific), 10% heat-inactivated fetal calf serum (Thermo Fisher Scientific), and 100 U/ml penicillin and 100 µg/ml streptomycin. Passaging of cells was carried out with TripLE dissociation reagent (Thermo Fisher Scientific).

### Proliferation analysis

Human CF and tsA201 cells were seeded in their respective growth media at a density of 25,000 cells/well into 24-well plates. Twenty-four hours after seeding (day 0), PFD dissolved in FGM-3 for human CF or in DMEM growth medium for tsA201 cells was added in the following concentrations: 0.1, 0.3, 1.0, or 3.0 mg/ml. The media including the treatments were changed every second day. The cells were fixed with 4% paraformaldehyde (PFA) in Dulbecco’s phosphate-buffered saline (DPBS) for 10 min on days 0, 2, and 4 (human CF) or days 0, 2, and 3 (tsA201). For nuclear staining, 1 µg/ml DAPI in DPBS was added for 30 min in the dark, followed by washing with DPBS. Automated evaluation of the cell number was assessed either by automated counting using the Cellavista System (human CF, SynenTec) or by measuring the relative immunofluorescence units with the FlexStation 3 multi-mode microplate reader (tsA201, Molecular Devices). The relative change in signal was calculated compared to day 0. The differences in signal compared to the last culture day were used to calculate the IC_50_ with the help of GraphPad Prism 8.

### Cell staining

For cell staining, either the fixed human CF from the proliferation assay at day 2 were taken, or 5000 cells/well were seeded into 24-well plates and let grown until they reached a confluency of around 50%. Then, PFD was added dissolved in FGM-3 in a concentration of 1.0 mg/ml together with 5 ng/ml TGF-β1, and the cells were fixed 48 h later with 4% PFA in DPBS for 5 min. The cells were permeabilized with 0.2% Triton X-100 in DPBS for 3 min and washed with DPBS. Blocking occurred in Roti-Immunoblock (Carl Roth) for 1 h. For α-smooth muscle actin staining, the antibody was diluted 1:1000 in DPBS with 0.1 × Roti-Immunoblock and incubated with the cells at 4 °C overnight. After washing with DPBS, the secondary Alexa Fluor 488-coupled antibody was added for 1 h in DPBS with 0.1 × Roti-Immunoblock at room temperature together with 1 µg/ml DAPI and a 1:60 dilution of Alexa Fluor 633-phalloidin. The incubation was carried out in the dark. The fluorescence staining was imaged with a CQ1 confocal imager (Yokogawa) and a 20 × long distance objective. Areas of 6 × 5 single images were recorded in 3 Z-planes (distance 6 µm) and the images were projected by maximum intensity via Z Project (ImageJ). Zoom-ins of the projections are also presented.

The fixed cells of the proliferation assay were incubated with TRITC-phalloidin (0.5 µg/ml), DAPI (1 µg/ml), and WGA-Alexa Fluor 488 (5 µg/ml) in DPBS. The staining was performed for 1 h at room temperature in the dark. Afterward, the cells were washed twice with DPBS and imaged with an inverted fluorescence microscope (Olympus) at a magnification of 20 × .

### Immunoblot analysis

To prepare samples for protein analysis, the cells were seeded in FGM-3 into 6-well plates at a density of 150,000 cells/well. After reaching a confluency of 80–90%, the cells were treated with 0, 0.3, or 1.0 mg/ml PFD dissolved in FGM-3 for 4 h in duplicates. Subsequently, one of the duplicates was incubated with 5 ng/ml TGF-β1 (Preprotech) for 30 min. The cells were washed with DPBS and lysed with ice-cold Cytobuster lysis buffer (Millipore) supplemented with protease and phosphatase inhibitors (Roche). The cell lysates were centrifuged for 30 min at 12,000 × g and 4 °C and the supernatants were subjected to a 12% SDS-PAGE. After the transfer onto nitrocellulose membranes (Amersham), the membranes were blocked in 1 × Roti-Block solution and incubated with the primary antibodies overnight at 4 °C. After three washing steps, the secondary antibodies were applied for 1 h at room temperature. Finally, the membranes were washed and the antibody complexes were detected using SuperSignal West Femto Maximum Sensitivity Substrate (Thermo Fisher Scientific) and a ChemiDoc MP Imaging System (Bio-Rad). The detection of the total protein amounts of the kinases was performed on membranes that have been used to detect the phosphorylated variants after incubating them in Roti-Free Stripping Buffer 2.2 plus (Carl Roth) for 1 h at room temperature.

### Generation of human engineered connective tissues

The engineered connective tissues (ECT) generation was performed as describe before (Santos et al. 2021). In brief, all required materials were pre-chilled, and all following steps were performed on ice. First, 3 mg/ECT bovine collagen type I (Collagen solutions) was mixed with 2 × DMEM and neutralized with 0.1 M NaOH. Then, resuspended 7.5 × 10^5^ human CF /ECT in FGM-3 were added and thoroughly mixed. The final volume per ECT was 180 µl. The cell-collagen mixture was pipetted in 48-well mold plates containing two flexible poles (non-uniform model, myrPlates myriaMed) or self-made molds containing a central rod (uniform model) (Santos et al. 2019, 2022) and let condensate for 1 h at 37 °C in a cell incubator. Afterward, FGM-3 supplemented with 0.3 or 1 mg/ml PFD was added. The ECT were cultured for 5 days, and the medium, including the additives, was changed every second day.

### Contraction analysis

ECT contraction was estimated based on pole deflection. The poles of the mold plate contained an optical brightener that allowed images to be taken under a UVA light. Images were taken every day. The distances between the poles were analyzed with ImageJ and changes in pole deflection were calculated for each ECT relative to the initial pole distance at day 0 (Santos et al. 2021).

### Compaction analysis

After 5 days, the ECT were imaged from the side and top with a Lumar.V12 stereo microscope (Zeiss). The diameters of the ECT were measured at several positions and the cross-sectional areas (CSA) were calculated assuming an elliptical shape of the tissues (Santos et al. 2021).

### Destructive tensile strength measurements and stress–strain analysis

To assess the biomechanical properties of the ECT, destructive tensile strength measurements were performed using a RSA-G2 rheometer (TA instruments). Therefore, the ECT were placed on two opposite hooks in an organ bath filled with DPBS tempered to 37 °C. Before starting the measurement, the force and the gap were set to zero. The tissues were then stretched with a constant linear rate of 0.03 mm/s until the point of rupture. To obtain stress–strain curves, the recorded forces were divided in Excel by the CSA to obtain stress values. Afterward, these values were plotted against the strain values calculated by the equation (L_total_-L_0_)/L_0_ in which L_0_ represents the initial gap between the upper and lower hook and L_total_ is the total gap at each point. To determine the Young’s modulus, the slope of the linear region of the stress–strain-curve, linear regression analysis was performed. Further important properties of the curves, like yield point strain (end of the elastic region), maximum stress (highest stress point), and ultimate strain (sudden drop in stress) were identified manually (Santos et al. 2021).

### Dissociation of ECT and determination of cell number and viability

ECT were dissociated with pre-warmed collagenase solution (2 mg/ml collagenase I and 20% FBS in calcium-containing DPBS). The tissues were incubated for 1–3 h at 37 °C. The supernatants were collected, and the remaining tissues were washed with DPBS (w/o calcium), then incubated in a dissociation solution containing accutase, 0.025% trypsin, and 20 µg/ml DNAse at 37 °C for 30 min. After mechanical dispersion, the cell suspensions were centrifuged at 100 × g for 10 min at 4 °C and the pellets were resuspended in ice-cold DPBS containing 5% FBS. The number of total cells and their viability were assessed by using the CASY TTC instrument (Roche).

### Flow cytometry analysis

The cell cycle analysis was performed by FACS with cells isolated from ECT. In brief, the isolated cells were resuspended in ice-cold 4% PFA centrifuged at 300 × g for 5 min at 4 °C, resuspended in 200 µl blocking buffer (DPBS containing 5% FBS, 1% BSA, 0.5% Triton-X 100), and permeabilized for 5 min at 4 °C. Then, 10 µg/ml Hoechst33342 was added for 30 min on ice in the dark. Before the analysis, the cells were washed, strained (70 µm), and measured using the LSRII SORP Cytometer. For analysis, Flowing software (flowingsoftware.btk.fi, Perttu Terho) was used, cell debris was excluded, and subsequently, cells in G0/G1, S, and G2/M phases were identified based on the Hoechst33342 signal. Once adjusted by control cells, the same gating strategy was applied for all samples.

### Statistical analysis

Experimental data are presented as means ± SEM. Data was analyzed by one- or two-way ANOVA with appropriate multiple parameter testing. Statistical calculations were carried out by using GraphPad Prism 8 or 9. Significance was assumed when *p* < 0.05.

## Results

### PFD inhibits the proliferation of human CF in 2D cultures in a concentration-dependent manner

**Fig. 1:**
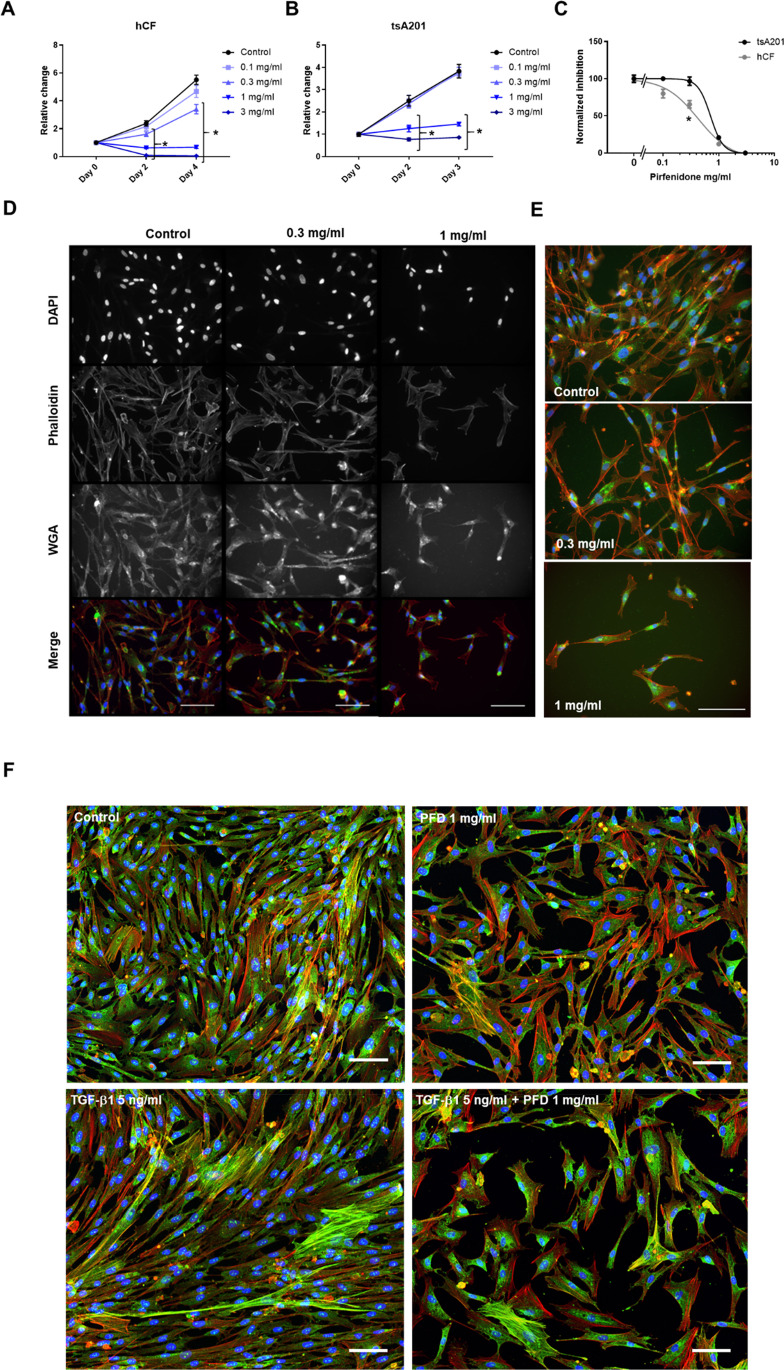
PFD inhibits human CF proliferation in 2D culture. **A** 2D-cultured human CF were treated with 0, 0.1, 0.3, 1.0, or 3.0 mg/ml PFD dissolved in FGM-3 for 0, 2, or 4 days. The cell number was assessed by DAPI staining and automatic counting. Given are the relative changes in cell number compared to day 0. All values are means ± SEM, *n* = 3–4 in 4 replicates. Statistical analysis was performed by a 2-way ANOVA with a Dunnett’s multiple comparison test vs. control, *p > 0.05. **B** 2D-cultured tsA201 cells were treated with 0, 0.1, 0.3, 1.0, or 3.0 mg/ml PFD dissolved in cultured in DMEM growth medium for 0, 2, or 3 days. Given are the relative changes in fluorescence intensity compared to day 0. All values are means ± SEM, *n* = 3–4 in 3 replicates. Statistical analysis was performed by a 2way-ANOVA with a Dunnett’s multiple comparison test vs. control, *p > 0.05. **C** The IC_50_ values of PFD were calculated by non-linear regression based on the differences detected at day 5 (human CF IC_50_ = 0.43 mg/ml, tsA201 IC_50_ = 0.71 mg/ml). Shown are the means ± SEM, *n* = 3–4. The comparison was performed by a 2way-ANOVA with a Sidak’s multiple comparison test, *p > 0.05. **D**, **E** The fixed human CF from day 2 of the proliferation assay were stained with DAPI (gray/blue), TRITC-phalloidin (gray/red), and WGA-Alexa Fluor488 (gray/green) to detect cell nuclei, F-actin, and glycolipids and -proteins, respectively. **D** All images were taken with the same magnification (scale bar 100 μm). The merges are presented in the last row. E) Merged images with higher magnification (scale bar 100 μm) are shown in addition. **F** 2D-cultured human CF were treated with or without 5 ng/ml TGF-β1 and 1 mg/ml PFD for 48 h. SMA (green) was detected by immunofluorescence. F-actin (red) and nuclei (blue) were stained in addition. The merges are presented with a scale bar of 100 µm

At first, the effect of PFD on the proliferation of human CF cultured in FGM-3 was analyzed. With lower PFD concentrations (0.1, 0.3 mg/ml), a decrease in the proliferation rate of human CF was observed. Concentrations above 1.0 mg/ml, however, led to a substantial cell loss (Fig. 1A). In contrast, tsA201 cells cultured in DMEM growth medium were not affected by lower PFD concentrations, but similar cytotoxic effects occurred with concentrations above 1.0 mg/ml (Fig. 1B). For better comparison, the differences on the last day of measurements were plotted as normalized values in one graph, demonstrating the higher sensitivity of human CF against PFD (Fig. 1C). From the concentration–response curves, IC_50_ values of 0.43 mg/ml for human CF and 0.71 mg/ml for tsA201 could be determined. In addition, the impact of PFD on human CF morphology was analyzed. Therefore, human CF were fixed after 2 days of PFD treatment and stained with DAPI, TRITC-phalloidin, and WGA-Alexa Fluor 488 to detect nuclei, F-actin, and glycosylation, respectively. Except for the expected decrease in cell number, no striking differences in actin fiber formation and glycosylation were observed. The nuclear and cell morphologies appeared to be unaltered (Fig. 1D, E).

To further analyze the effect of 1 mg/ml PFD on human CF, we added 5 ng/ml TGF-β1 in the presence and absence of PFD to the FGM3 medium and cultured the cells for 48 h. The co-staining of SMA with Alexa Flour 633-phalloidin for F-actin and DAPI demonstrated that the density of human CF was moderately increased in the presence of TGF-β1 (Suppl. Figure 1). Importantly, more cells displayed F-actin fibers with incorporated SMA. As described before, PFD application inhibited cell proliferation in the absence of TGF-β1 and also clearly in the presence of it. Cells with SMA-containing F-actin fibers were in both cases still detectable, although they appeared to be moderately reduced in number (Fig. 1F, Suppl. Figure 1). Of note, in all conditions, a diffuse SMA background staining with a similar intensity could be detected, which have been already described by others to be present in proto-myofibroblasts (Hinz et al. 2007; Hillsley et al. 2021).

### PFD inhibits the phosphorylation of members of the MAPK pathways in 2D-cultured human CF

**Fig. 2:**
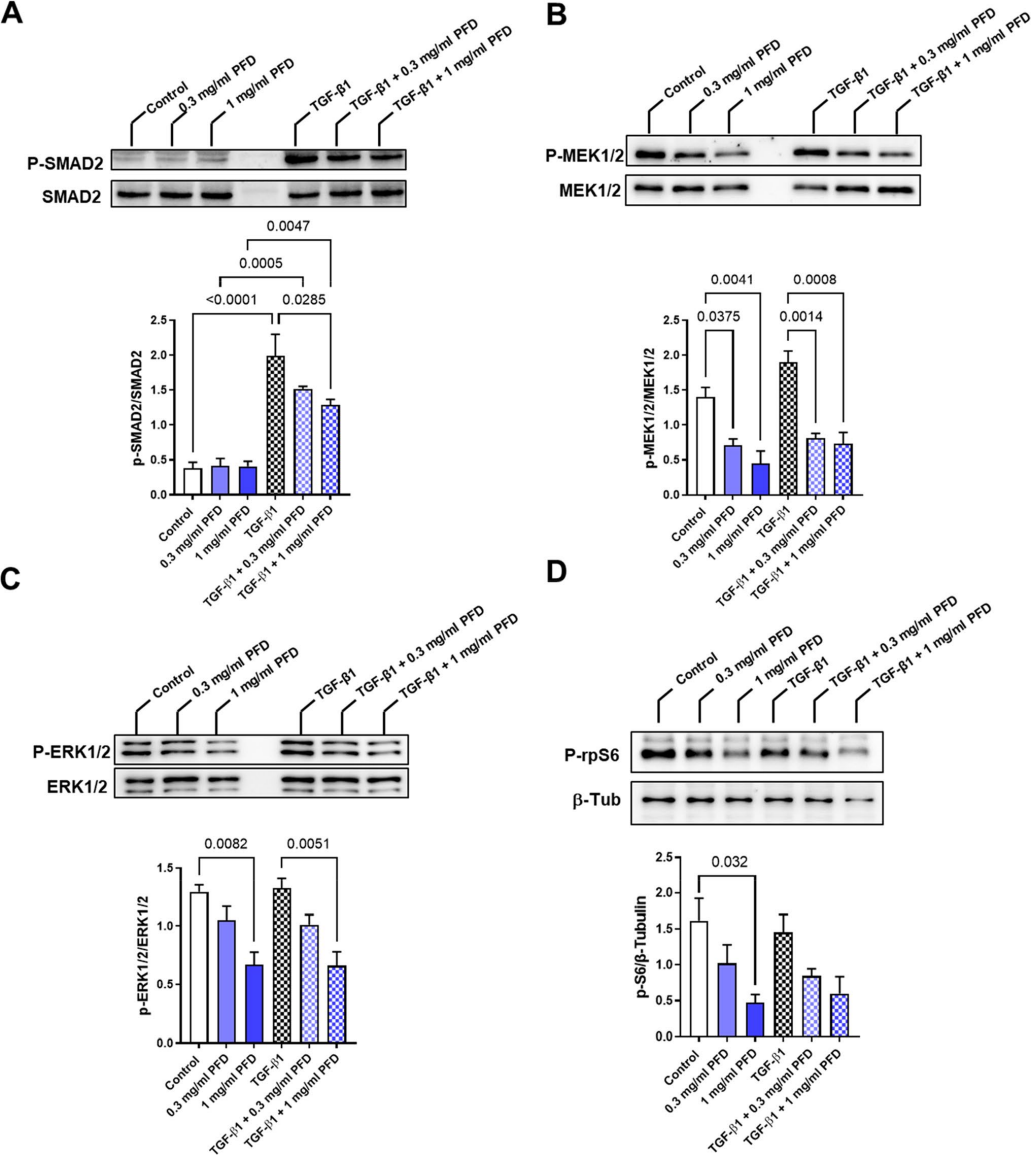
PFD prominently inhibits the MAPK pathway in 2D-cultured human CF. Human CF were treated with 0, 0.3, or 1.0 mg/ml PFD in FGM-3 for 4 h and then 5 ng/ml TGF-β1 was applied for 30 min. Cell lysates were used for immunoblot analysis. Shown are representative immunoblots and the analysis of **A** SMAD2, **B** MEK1/2, **C** ERK1/2, and **D** rpS6 phosphorylation. All results were obtained from 3 independent experiments and shown are the means + SEM with the significant *p* values as assessed by 1way-ANOVA with Tukey’s multiple comparison test

To assess if PFD interferes with the canonical TGF-β1 signaling in human CF, confluent cell cultures of human CF were pre-incubated with 0, 0.3, or 1.0 mg/ml PFD in FGM-3 for 4 h before 5 ng/ml TGF-β1 was added for 30 min. The lysates were used for immunoblot analysis (Suppl. Figure 2). The results demonstrated that TGF-β1 increased the phosphorylation of SMAD2 more prominently than of SMAD3 and PFD pre-treatment reduced only SMAD2, but not SMAD3 phosphorylation (Fig. 2A, Suppl. Figure 3A). Based on the anti-proliferative activity of PFD, the MAPK pathway was studied in addition. The analysis revealed that TGF-β1 had no effect on MEK1/2, ERK1/2, and rpS6 phosphorylation. However, PFD reduced the phosphorylation of MEK1/2, ERK1/2, and rpS6 under basal conditions as well as in the presence of TGF-β1 in a concentration-dependent manner (Fig. 2B–D). For further analysis, we used an antibody that recognizes phosphorylated proteins with the Akt substrate sequence RXXS*/T* and detected a PFD-sensitive phospho-protein, which displayed a similar size and phosphorylation pattern as rpS6 (Suppl. Figure 3B).

### PFD inhibits compaction and contraction of ECT

The effect of PFD on the viability of 3D-cultured human CF and their impact on the biomechanical properties of ECT have not been studied before. Further, it is not known if PFD affects CF with different phenotypes differentially. Therefore, we generated ECT in molds with different geometries as we have previously shown that the mechanical constraint and consequently the cellular reaction to it is largely dependent on the mold shape. In brief, when the ECT forms around a central rod (uniform model), the equal and high mechanical stress induces a stronger myofibroblast phenotype compared to when the ECT forms around two distant poles (non-uniform model). Consequently, pro-fibrotic gene expression and ECT stiffness is higher in the uniform compared to the non-uniform model. Moreover, we found that cell survival is improved in the uniform ECT (Santos et al. 2022).

**Fig. 3:**
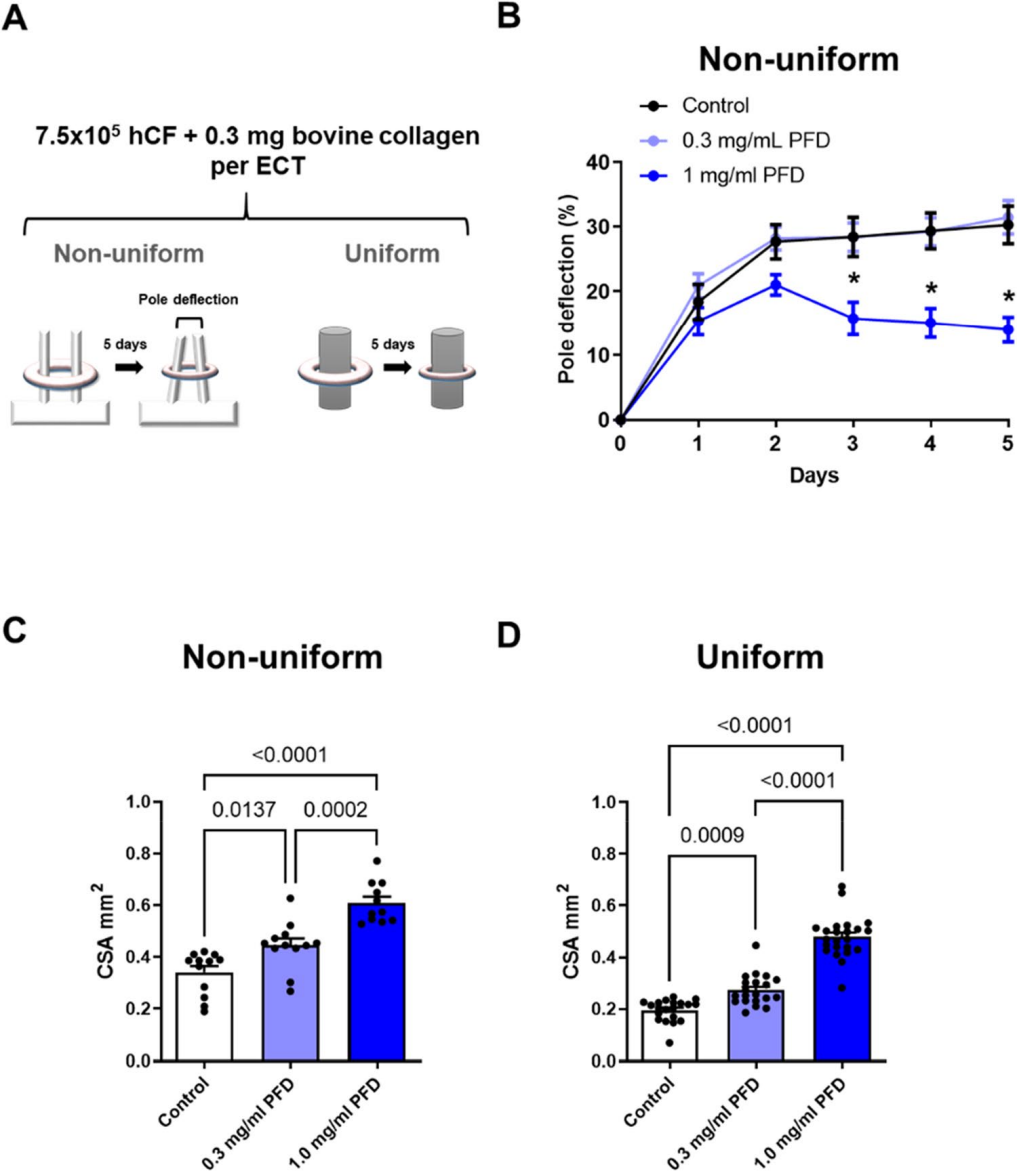
PFD inhibits ECT compaction and contraction. **A** 7.5 × 10^5^ human CF (hCF) together with 0.3 mg bovine collagen I were used to generate ECT in molds equipped with either two flexible poles (non-uniform model) or a central rod (uniform model). ECT were treated 1 h after generation with 0, 0.3, or 1 mg/ml PFD dissolved in FGM-3 for 5 days. **B** Pole deflection was assessed over a period of 5 days in the non-uniform model and is given as percent change compared to day 0. The data represents means ± SEM of 18 ECT per group, *p < 0.05, *compared to control and to 0.3 mg/ml PFD, assessed by 2-way ANOVA with Tukey’s multiple comparison test. **C**, **D** The CSA was analyzed from macroscopic images of non-uniform and uniform ECT. The bars represent the means + SEM and the values of the individual ECT (*n* = 11–23) are shown as symbols, *p* values were assessed by 1-way ANOVA with Tukey’s multiple comparison test

To prepare non-uniform and uniform ECT, we used for both types 7.5 × 10^5^ human CF and 0.3 mg bovine collagen I per ECT, started the treatment with 0.3 or 1 mg/ml PFD 1 h after casting, and cultured them for 5 days. During this period, pole deflection was assessed every day in the non-uniform model to estimate ECT contraction. After the first day, all ECT showed a nearly similar contraction. From day 3 on, the contraction levels of the control and the lower PFD treatment group (0.3 mg/ml) reach almost a plateau. On the contrary, the group with the higher PFD treatment (1.0 mg/ml) was by trend lower on day 2 and showed in the following an impairment in contraction. This resulted in an almost 50% reduced pole deflection at day 5 compared to the control and lower PFD treatment groups (Fig. 3B). In addition to ECT contraction, the ability of the cells to compact the collagen matrix was studied in both models. Therefore, macroscopic images from different positions were taken and the CSA were calculated with the help of the measured diameters and by assuming an elliptical tissue cross section. Thereby, we could verify the enhanced compaction of ECT in the uniform versus non-uniform model (Santos et al. 2022) and importantly we showed that the PFD treatment resulted in a concentration-dependent increase in the CSA in both models (Fig. 3C, D).

### PFD reduces the number and viability of embedded human CF in ECT and inhibits cell cycle activity

**Fig. 4:**
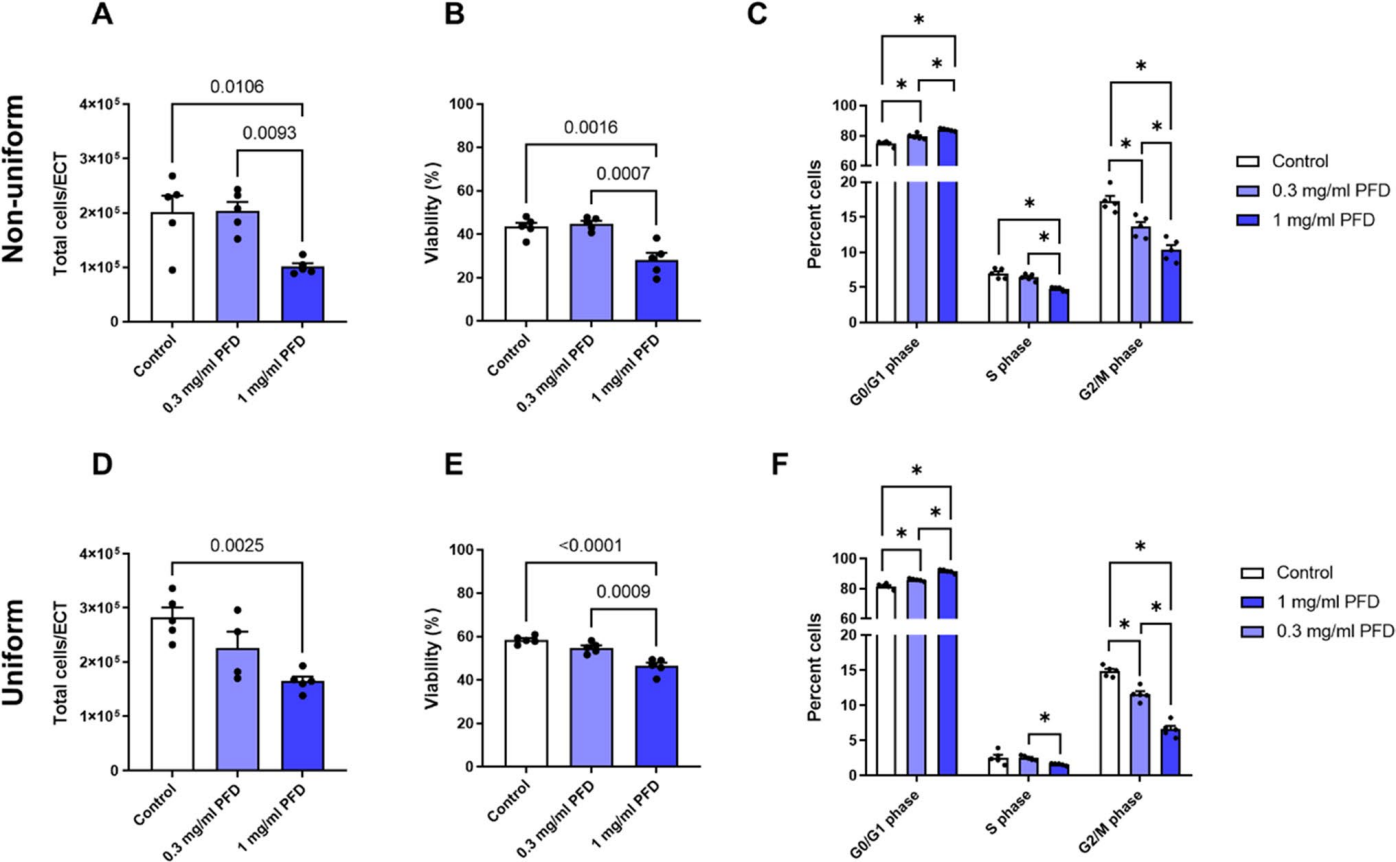
PFD impairs cell viability and cell cycle activity of CF in ECT. ECT were generated in molds with two flexible poles (non-uniform) or a central rod (uniform) and treated 1 h after generation with 0, 0.3, or 1 mg/ml PFD dissolved in FGM-3 for 5 days. Cell isolation was carried out by collagenase/accutase digest. **A**–**E** Cell numbers and viability were analyzed with a Casy TTC system. The bars represent the means + SEM and the values of the individual ECT (*n* = 4–5) are shown as symbols, p-values were assessed by 1-way ANOVA with Tukey’s multiple comparison test. **C**, **F** The isolated cells were fixed and stained with Hoechst33342. FACS analysis was carried out. The bars represent the means + SEM and the values of the individual ECT (*n* = 5) are shown as symbols, *p > 0.05, assessed by 2-way ANOVA with Tukey’s multiple comparison test

To study the effect of PFD on the number and viability of the embedded cells after 5 days of culture, we isolated them by a sequential collagenase-accutase digest and analyzed their number and viability by electric current exclusion. As already described before, a loss of cells during the 5 days of culture was observed in all conditions and this loss was more pronounced in non-uniform ECT (Santos et al. 2022). Importantly, the application of 1 mg/ml PFD resulted in a more pronounced cell loss (Fig. 4A, D) and reduced cell viability in both ECT models when compared to the control (Fig. 4B, E). The isolated cells were further used to determine their cell cycle activity by FACS (representative gating is presented in Suppl. Figure 4). In both culture models, more cells were found in G0/G1 and less in S- and G2/M-phases in the presence of 1 mg/ml PFD compared to the control (Fig. 4C, F).

### PFD treatment reduces the stiffness of ECT

**Fig. 5:**
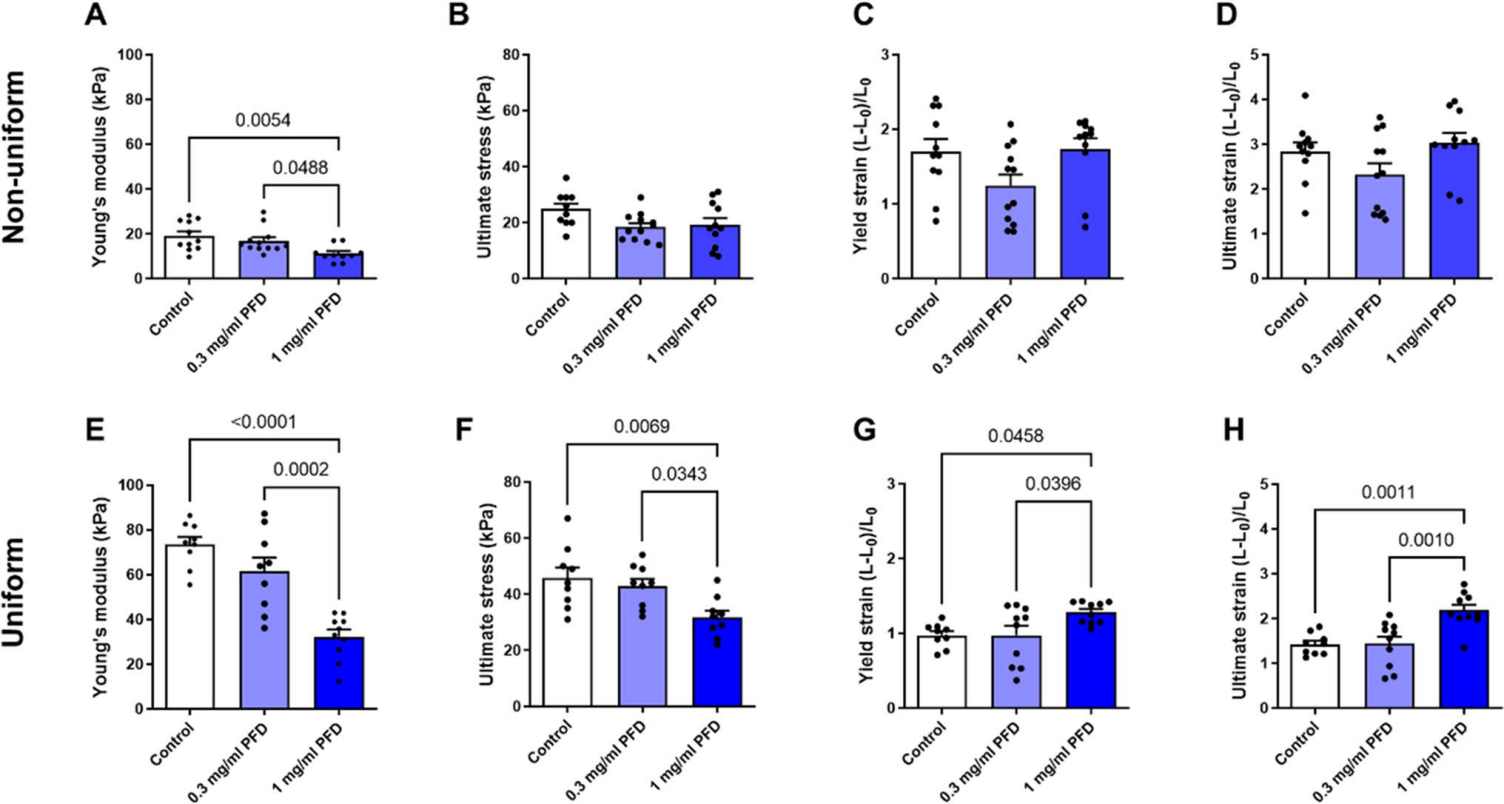
PFD reduces ECT stiffness of non-uniform and uniform ECT and increases strain resistance of uniform ECT. ECT were generated in molds with two flexible poles (non-uniform) or a central rod (uniform) and treated 1 h after generation with 0, 0.3, or 1 mg/ml PFD dissolved in FGM-3 for 5 days. Ultimate tensile testing was performed. **A**, **E** ECT stiffness (Young’s modulus), **B**, **F** strength (ultimate stress), **C**, **G** elasticity (yield strain), and **D**, **H** extensibility (ultimate strain) were determined based on the obtained stress–strain curves. The bars represent the means + SEM and the values of the individual ECT (*n* = 9–12) are shown as symbols, *p* values were assessed by 1way-ANOVA with Tukey’s multiple comparison test

Finally, we wanted to know if PFD influences the biomechanical properties of the ECT. Therefore, we performed an ultimate tensile test with a dynamic mechanical analyzer. From the obtained stress–strain curves, important biomechanical parameters were retrieved. We found that 1 mg/ml PFD reduced the ECT stiffness in the non-uniform and uniform model (Fig. 5A, E), as well as the ultimate stress in the uniform model (Fig. 5F). Furthermore, the elasticity and extensibility of the uniform ECT, as reflected by the yield and ultimate strains, respectively, were improved (Fig. 5G, H). Taken together, PFD displayed beneficial effects on the biomechanical tissue properties, especially in the uniform model.

## Discussion

Progressive cardiac fibrosis is a major complication in heart disease and new efficacious anti-fibrotic drugs are urgently needed. Drug repurposing offers a possibility to accomplish this task faster. One example is PFD, which has been used for the treatment of IPF in Europe since 2011 and is now being tested for its anti-fibrotic action in patients with HFpEF. First published results with 39 patients in the treatment group showed a 1.2% reduction in extracellular volume after 52 days of treatment (Lewis et al. 2021).

Despite this significant result, considering that anti-fibrotic drugs for general use with every fibrotic disease might be critical, because it ignores tissue- and cell type-specific characteristics. Therefore, more basic research regarding the effects of PFD on human cardiac cells is necessary, especially as the specific mechanism of action of PFD is still unclear. One prominent example, which underlines the need for more research on human cells, is the reported consistent and inconsistent regulation of the myofibroblast marker SMA by PFD in human lung and cardiac fibroblasts, respectively (Molina-Molina et al. 2018; Jin et al. 2019; Palano et al. 2020; Widjaja et al. 2021; Bracco Gartner et al. 2022).

Initially, we were also interested in the PFD-dependent regulation of fibrosis-associated gene expression in human CF; however, in preliminary experiments, we could not detect substantial changes in SMA and pro-collagen expression by immunoblot analysis (data not shown). A possible explanation for this is given by the SMA immunostaining in Fig. 1. It clearly shows that PFD does not reduce the diffuse SMA staining, which marks activated CF or proto-myofibroblasts, but might moderately reduce myofibroblasts characterized by SMA-containing stress fibers. Thus, the mere detection of SMA is not appropriate to judge the phenotype of CF. More sophisticated imaging methods are needed to classify these heterogeneous cells (Hillsley et al. 2021).

Based on the somehow disappointing effects of PFD on the expression of fibrosis-associated factors, we focused on the not yet investigated anti-mitogenic effect of PFD in human CF. By a concentration–response analysis, we determined an anti-proliferative IC_50_ of 0.43 mg/ml for PFD for human CF cultured in regular growth medium, which is in a similar range as demonstrated for rat CF and other fibroblast types (Lin et al. 2009; Shi et al. 2011; Tao et al. 2020). Moreover, we found that 1 mg/ml PFD resulted in cell number stagnation and 3 mg/ml eliminated all seeded human CF within 2 days. We further show that 1 mg/ml PFD inhibited cell proliferation when TGF-β1 was present. For IC_50_ comparison, we used highly proliferative, immortalized tsA201 cells, and found that these cells were slightly less sensitive to PFD, as indicated by the higher IC_50_ and lower cytotoxicity. This data indicates that not all cell types display the same sensitivity to PFD, which could explain, e.g., the discrepancy between published cytotoxic PFD concentrations (Shi et al. 2011; Mediavilla-Varela et al. 2016).

We further investigated the effect of PFD on the TGF-β1-induced SMAD signaling, as well as on the central mitogenic MEK1/2-ERK1/2 cascade. We found that PFD was without effect on basal SMAD phosphorylation and inhibited moderately SMAD2, but not SMAD3 phosphorylation in the presence of TGF-β1. In contrast, MEK1/2 and ERK1/2 phosphorylation was prominently reduced under basal conditions, indicating that the mitogenic signaling is more sensitive to PFD than the basal pro-fibrotic signaling in the 2D-cultured myofibroblasts. Of note, we could not detect any significant influence on the phosphorylation of both kinases by TGF-β1 as demonstrated by others for ERK1/2. We believe that this is not at least due to different experimental conditions, which could include media composition, serum starvation, TGF-β1 concentration, treatment duration, cell passage number, and cell phenotype. For example, Widjaja and colleagues demonstrated that TGF-β1 elicited a biphasic ERK1/2 phosphorylation in serum-starved human CF with a milder response after 15 min and a stronger after 24 h. The PFD effect on ERK1/2 phosphorylation was shown after 24 h (Widjaja et al. 2021). We, in contrast, did not serum-starve and analyzed the PFD effects after 15 min.

We further show that PFD inhibits the phosphorylation of rpS6, which is a downstream target of the MEK1/2-ERK1/2 pathway and phosphorylated by p90 ribosomal S6 kinases (RSK) (Carriere et al. 2008). Alternatively, an inhibition of Akt/mTor signaling by PFD could be the cause for the reduced rpS6 phosphorylation via S6-kinase 1 (Li et al. 2018). Although, we did not directly test for Akt phosphorylation, we used an antibody that detects proteins phosphorylated at the motif RXXS*/T*, which is also targeted by Akt. By this, we identified a regulated protein with signal characteristics similar to P-rsP6, but also other phospho-proteins appeared to be affected. Although the role of rpS6 phosphorylation is still not fully clear, it is discussed to play a role in mRNA translation, determination of cell size, and glucose homeostasis (Ruvinsky et al. 2005; Bohlen et al. 2021); further studies might investigate if one of these processes is impaired by PFD. Moreover, it would be interesting to see, if other kinases involved in proliferation and survival including p38 and c-Jun N-terminal kinases are also impaired by PFD in human CF as already shown for other human fibroblasts in the presence and/or absence of TGF-β1 (Conte et al. 2014; Guo et al. 2017; Haak et al. 2017; Hall et al. 2018; Shi et al. 2019; Zhou et al. 2019; Wu et al. 2021).

In addition to our 2D proliferation analysis, we studied the effect of PFD on the cell number, viability, and cell cycle activity of human CF in 3D cultures. Importantly, we were interested if the PFD effect is dependent on the CF phenotype and treated therefore non-uniform and uniform ECT, which differ in the phenotypic adaptation of the embedded cells during culture. As shown before, the uniform geometry induces a significantly more pronounced myofibroblast phenotype and a better cell survival compared to the non-uniform geometry (Santos et al. 2022). Importantly, we found that PFD treatment affected the cell number, viability, and cell cycle activity of human CF in both models to a similar extent. We show that 1 mg/ml PFD further reduced the cell number by 40 to 50% in both models and cell viability as well as cell cycle activity were similarly impaired, suggesting that PFD does not act differentially on different CF phenotypes. This also indicates that the cytotoxic effect of PFD appears to be more prominent in cases where the rate of cell death surpasses cell proliferation as normally seen in 3D, but not in 2D culture (Santos et al. 2022). We further believe that the sudden stagnation in non-uniform ECT contraction after 2 days of treatment with PFD is not a sign of an impaired contractile ability of the embedded cells, but a consequence of the enhanced cell loss. We have shown before that inhibitors acting directly on the cell’s contractile machinery, like Latrunculin A, reduce ECT contraction immediately and continuously and not delayed and abruptly as found for PFD (Santos et al. 2021). Similarly, the PFD-dependent inhibition of ECT compaction and stiffening might be a consequence of the enhanced loss of cells. Thus, our data indicate that the anti-fibrotic effects of PFD in our ECT model merely resulted from its anti-proliferative and/or cytotoxic activity. Moreover, PFD effects were relatively similar in both of our ECT models, and together with the demonstrated anti-proliferative activity in an immortalized cell line, its action on proliferating cells can be considered highly non-cell type-specific. Thus, it is not surprising that PFD is nowadays also discussed as a potential anti-cancer therapeutic (Paliogiannis et al. 2021).

The unspecific inhibition of cell proliferation together with the low potency of PFD and its very simple chemical structure raises the question of whether PFD has one distinct target or acts in a more general way. In this context, PFD was described to interfere with the redox system (Salazar-Montes et al. 2008; Fois et al. 2018; Sun et al. 2018) and that this might exert non-cell type-specific effects on fundamental cellular functions, like the herein demonstrated anti-proliferative/cytotoxic effect.

## Conclusion and perspective

Although PFD treatment is established in the therapy of IPF and was found to reduce the relative risk of mortality (Nathan et al. 2017), the disadvantages of this drug must be considered in other settings. First, IPF is a rapidly progressing disease with a mean survival time of 3 to 5 years (Spagnolo et al. 2021). Heart conditions with signs of fibrosis often show a slower progression than IPF, and thus anti-fibrotic therapy would be necessary for a longer duration, but long-term adverse effects of PFD are largely unknown. Second, not knowing a drug’s mechanism of action makes it difficult to predict adverse effects or interactions in certain situations. For example, among the common side effects of PFD is a transient increase in liver enzymes without clinical complications; however, under so far unknown conditions, PFD was recently found to induce acute liver injury, which could be rarely even fatal (Verma et al. 2018). In this context, it must be considered that the collective of patients that would need PFD to prevent cardiac fibrosis is substantially higher than that with the rare diagnosis IPF. And finally, as also demonstrated herein, PFD shows a highly non-selective anti-proliferative activity, which could potentially affect in the long term other processes relying on cell proliferation.

Taken together, PFD might be beneficial to a certain extent in the setting of cardiac fibrosis as the PIROUETTE trial suggests; however, the development of new, potent, and targeted anti-fibrotic drugs would be preferable.

## Supplementary Information

Below is the link to the electronic supplementary material.

Supplementary file1 (DOCX 6248 KB)

## Data Availability

The authors confirm that the data supporting the findings of this study are available within the article and its supplementary materials.
