# Supplementary material for: Pirfenidone affects human cardiac fibroblast proliferation and cell cycle activity in 2D cultures and engineered connective tissues
Source: Naunyn Schmiedebergs Arch Pharmacol. 2023 Feb 17;396(8):1687–99. doi: 10.1007/s00210-023-02421-9 (PMC10338590; doi:10.1007/s00210-023-02421-9)
Supplement: Supplementary file 1 — Supplementary file1 (DOCX 6248 KB) [file 210_2023_2421_MOESM1_ESM.docx]

**Suppl. Figure 1**

**
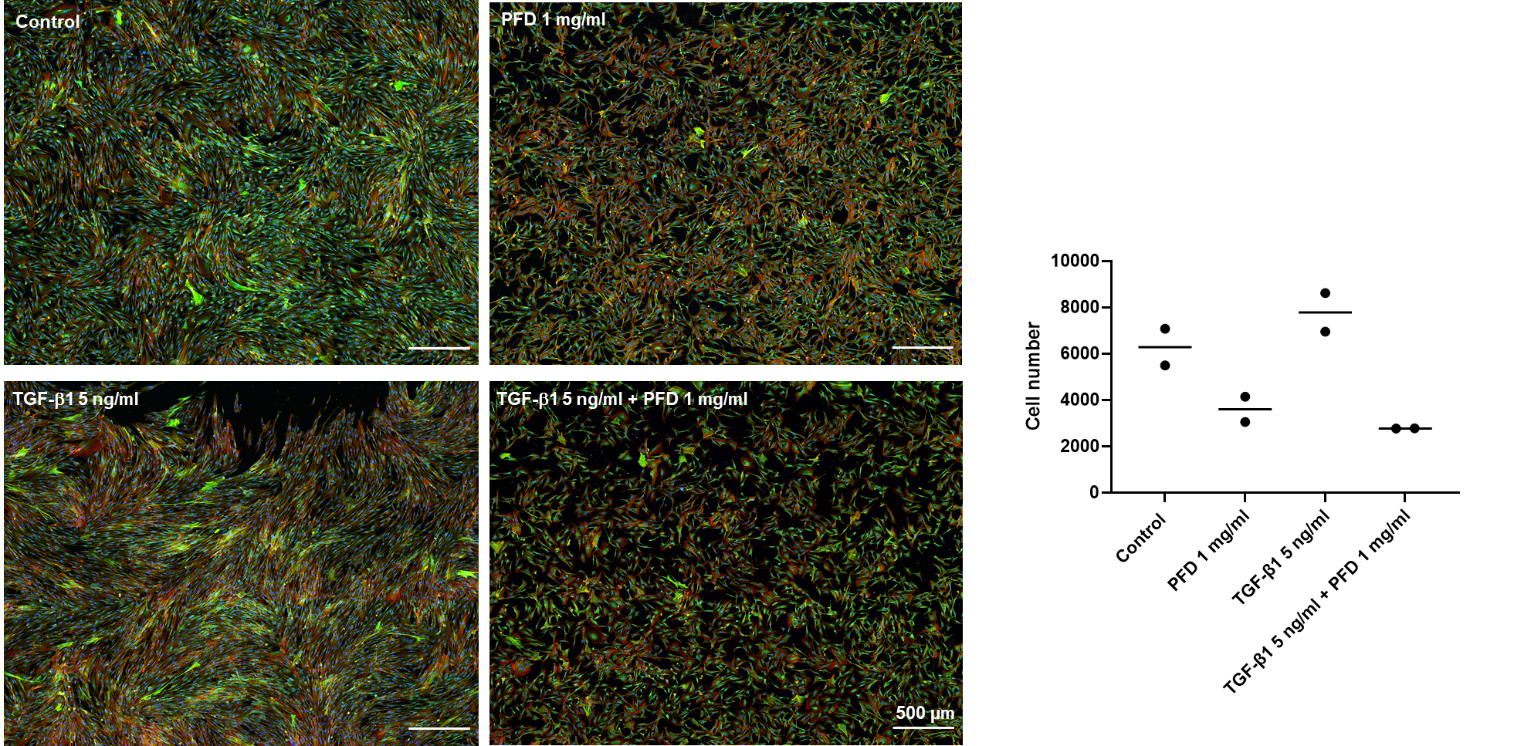
**

**Suppl. Figure 1: PFD inhibits proliferation of 2D-cultured human CF in the absence and presence of TGF-β1.** 2D-cultured human CF were treated with or without 5 ng/ml TGF-β1 and 1 mg/ml PFD for 48 h. SMA (green) was detected by immunofluorescence. F-actin (red) and nuclei (blue) were stained in addition. Left: Areas of 6x5 single images were recorded in 3 Z-planes (distance 6 µm) and the images were stitched together and projected by maximum intensity via Z Project (Image J). Scale bar 500 µm. Right: The nuclei were automatically counted in the complete area of 6x5 images of 2 separate wells per condition.

**Suppl. Figure 2**

**A**


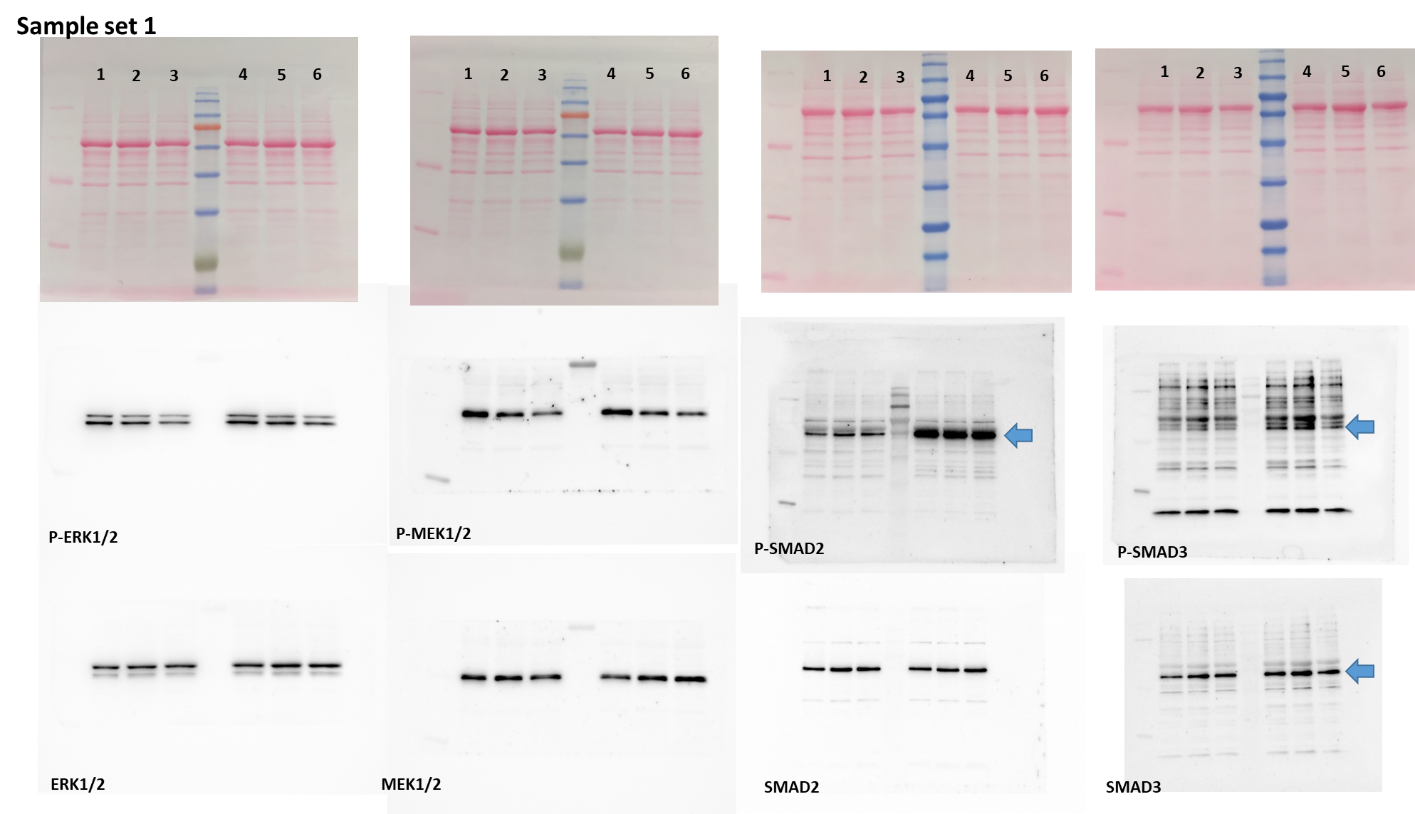


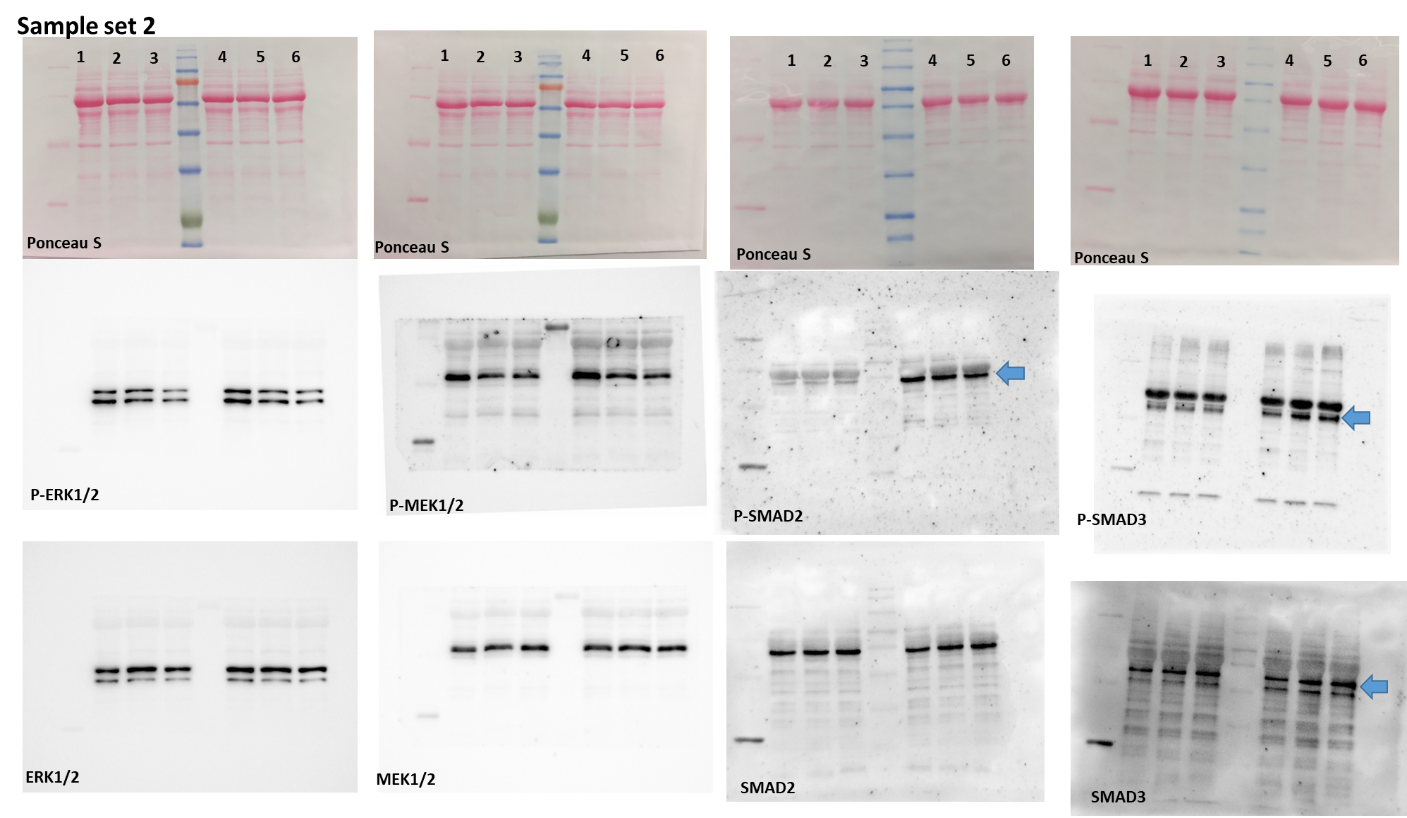


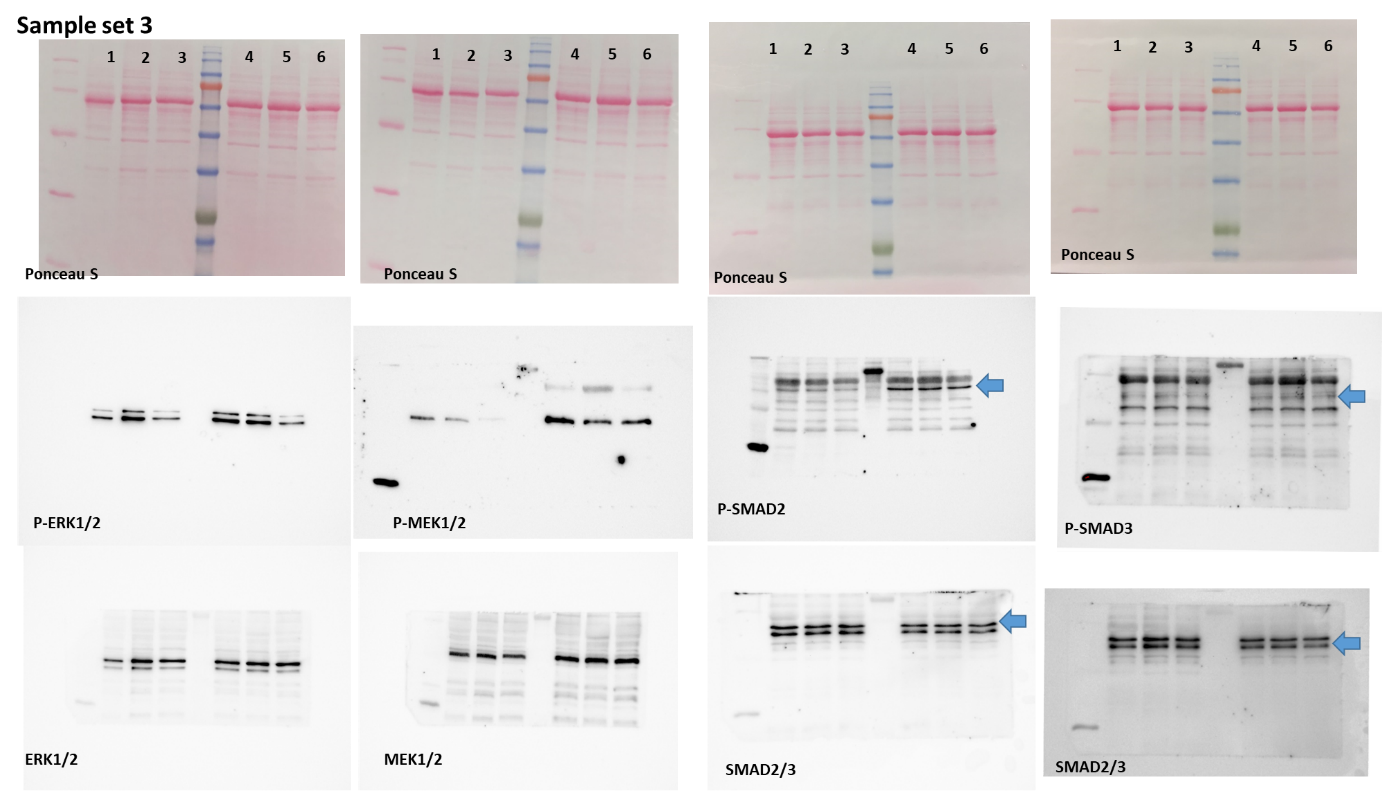


**B**


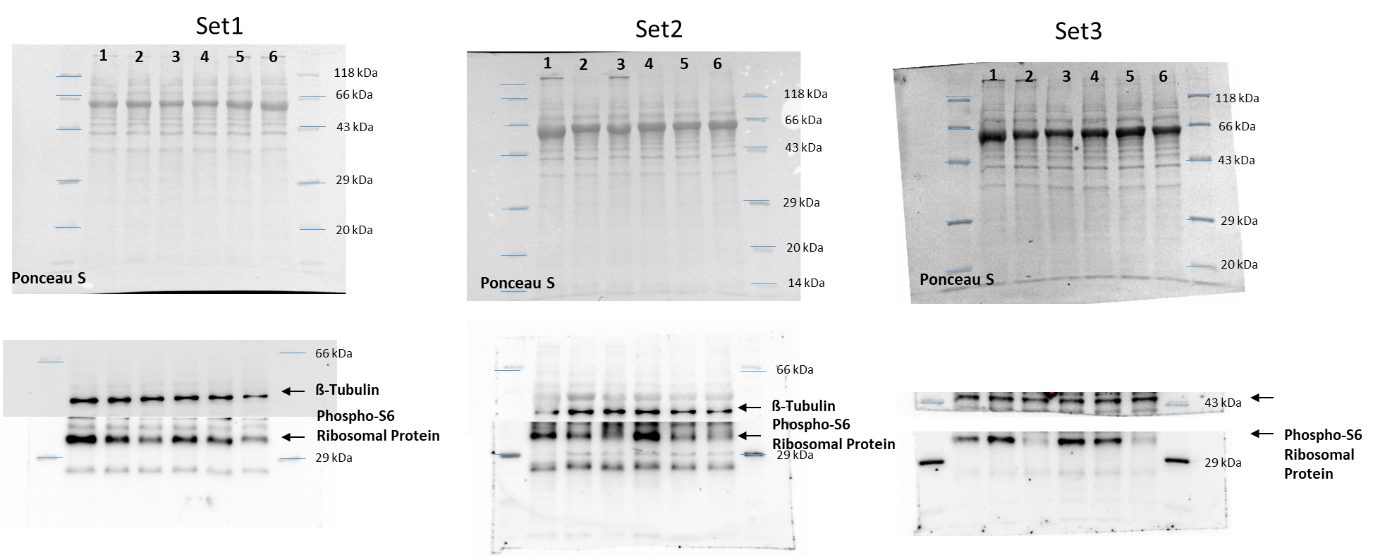


**C**


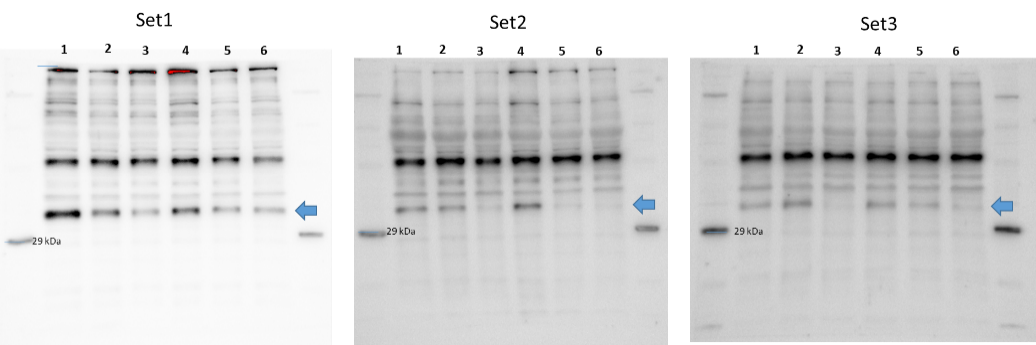


**Suppl. Figure 2: Raw images of Ponceau S staining and immunoblots.** Human CF were treated with 0, 0.3 or 1.0 mg/ml PFD for 4 h and then 5 ng/ml TGF-β1 was applied for 30 min. Cell lysates were used for immunoblot analysis. 1: Control, 2: 0.3 mg/ml PFD, 3: 1 mg/ml PFD, 4: 5 ng/ml TGF-β1, 5: 5 ng/ml TGF-β1 + 0.3 mg/ml PFD, 6: 5 ng/ml TGF-β1 + 1 mg/ml PFD. A) All ERK1/2, MEK1/2, SMAD2, and SMAD3 blots are given. B) β-Tubulin and P-rpS6 immunoblots are shown. C) The staining with the Akt substrate antibody (RXXS*/T*) is presented. Arrows indicate the analyzed bands.

**Suppl. Figure 3**

**
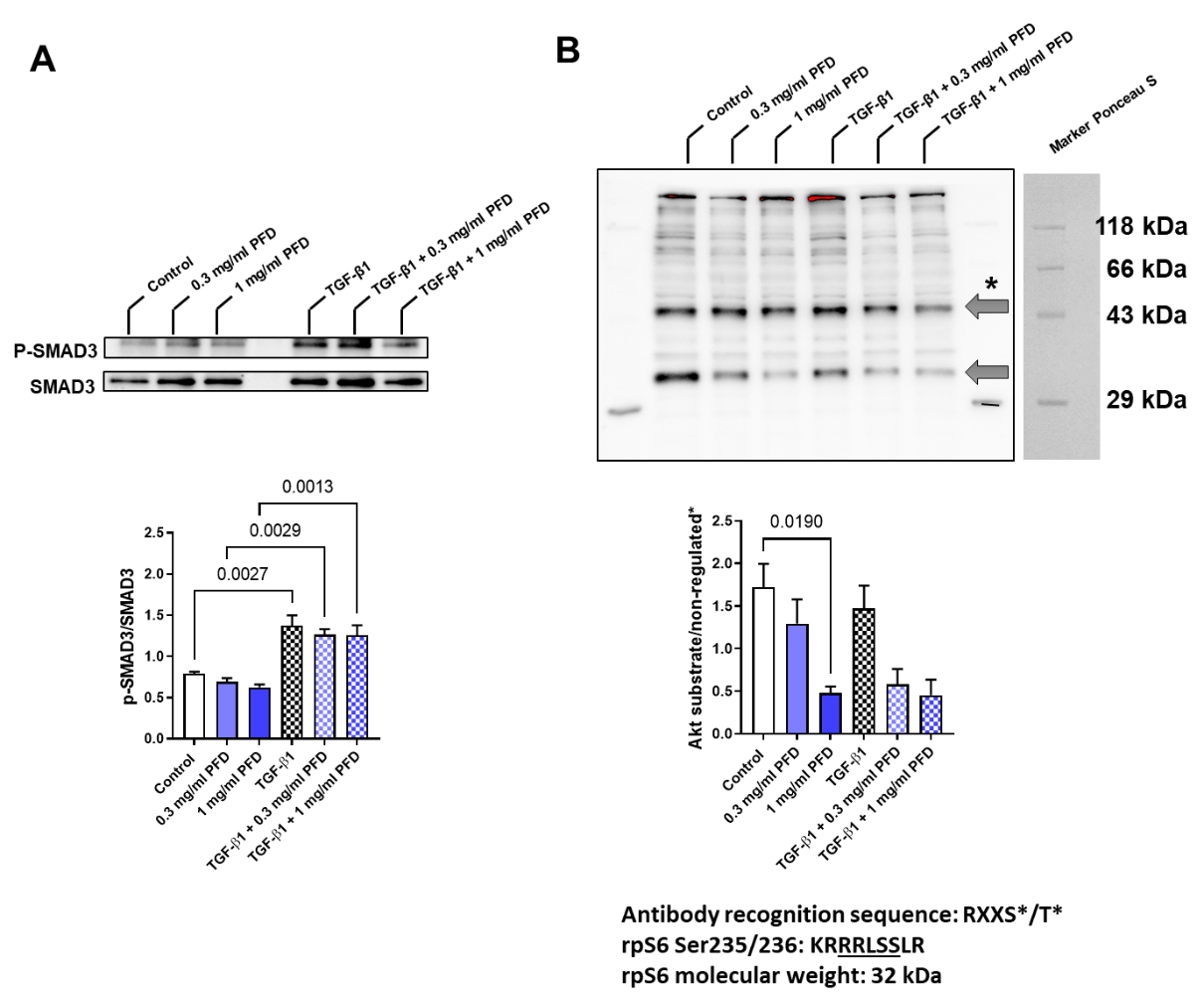
**

**Suppl. Figure 3: PFD effects on protein phosphorylation in 2D-cultured human CF.** **A)** Human CF were treated with 0, 0.3 or 1.0 mg/ml PFD for 4 h and then 5 ng/ml TGF-β1 was applied for 30 min. Cell lysates were used for immunoblot analysis. **A)** Shown are representative immunoblots and the analysis of SMAD3 phosphorylation. **B)** An antibody, which recognizes the phosphorylated sequence RXXS*/T* was used for the presented immunoblot and its analysis. The lower band, indicated by an arrow, is supposedly the phosphorylated version of rpS6. The upper indicated band was used for normalization. All results were obtained from 3 independent experiments and shown are the means+SEM with the significant p-values as assessed by 1way-ANOVA with Tukey’s multiple comparison test.

**Suppl. Figure 4**


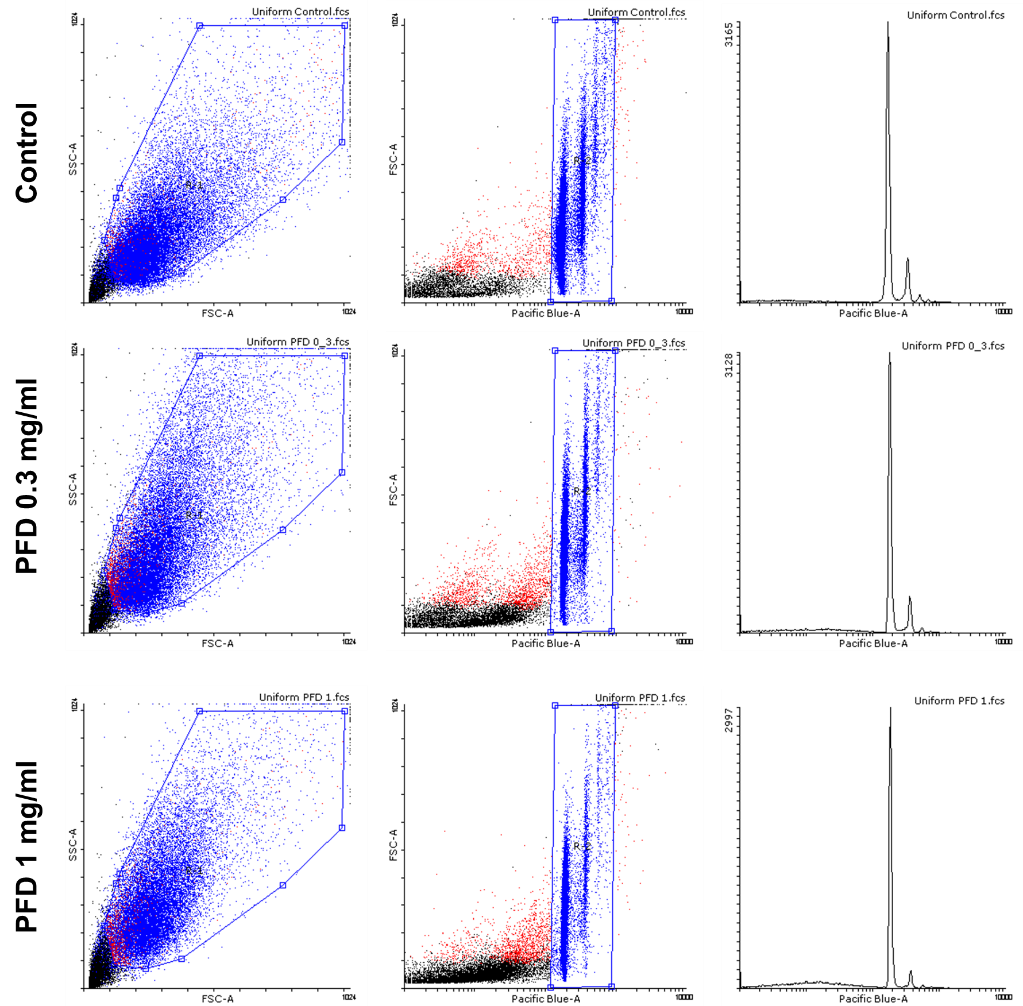


**Suppl. Figure 4: PFD effects on cell cycle activity of 3D-cultured human CF.** Representative FACS data of isolated human CF from uniform ECT, which were treated with 0, 0.3, or 1 mg/ml PFD for 5 days is shown.
